# MicroRNA-31 Reduces the Motility of Proinflammatory T Helper 1 Lymphocytes

**DOI:** 10.3389/fimmu.2018.02813

**Published:** 2018-12-06

**Authors:** Markus Bardua, Claudia Haftmann, Pawel Durek, Kerstin Westendorf, Antje Buttgereit, Cam Loan Tran, Mairi McGrath, Melanie Weber, Katrin Lehmann, Richard Kwasi Addo, Gitta Anne Heinz, Anna-Barbara Stittrich, Patrick Maschmeyer, Helena Radbruch, Michael Lohoff, Hyun-Dong Chang, Andreas Radbruch, Mir-Farzin Mashreghi

**Affiliations:** ^1^Deutsches Rheuma-Forschungszentrum (DRFZ), Berlin, Germany; ^2^Institute for Medical Immunology, Charité-Universitätsmedizin, Berlin, Germany; ^3^Department of Neuropathology, Charité-Universitätsmedizin, Berlin, Germany; ^4^Institute for Medical Microbiology and Hospital Hygiene, University of Marburg, Marburg, Germany

**Keywords:** CD4, miR-31, miRNA, target identification, T cell migration, Th1 cells, regulatory networks, antagomirs

## Abstract

Proinflammatory type 1 T helper (Th1) cells are enriched in inflamed tissues and contribute to the maintenance of chronic inflammation in rheumatic diseases. Here we show that the microRNA- (miR-) 31 is upregulated in murine Th1 cells with a history of repeated reactivation and in memory Th cells isolated from the synovial fluid of patients with rheumatic joint disease. Knock-down of miR-31 resulted in the upregulation of genes associated with cytoskeletal rearrangement and motility and induced the expression of target genes involved in T cell activation, chemokine receptor– and integrin-signaling. Accordingly, inhibition of miR-31 resulted in increased migratory activity of repeatedly activated Th1 cells. The transcription factors T-bet and FOXO1 act as positive and negative regulators of T cell receptor (TCR)–mediated miR-31 expression, respectively. Taken together, our data show that a gene regulatory network involving miR-31, T-bet, and FOXO1 controls the migratory behavior of proinflammatory Th1 cells.

## Introduction

Chronic synovial inflammation in rheumatoid arthritis (RA) is dependent on the migration and retention of T cells (1). Proinflammatory type 1 Th (Th1) cells are particularly enriched in the inflamed joints of patients with RA (2, 3). These cells express the transcription factor TWIST1, a hallmark of Th1 cells which have undergone repeated rounds of reactivation (4). TWIST1 limits inflammation (4) and, at the same time, promotes the survival of Th1 cells in inflamed tissues by up-regulating the microRNA (miR)-148a which targets the pro-apoptotic protein Bim (5, 6). This is in accordance with the observation that T cells isolated from inflamed joints are resistant to apoptosis (7) and persist in inflamed tissues despite state-of-the-art immunosuppressive therapies (4).

While trafficking of proinflammatory Th1 cells to sites of inflammation is well-characterized [reviewed in Mellado et al. (1)], the molecular mechanisms mediating their retention within inflamed tissues remain unclear. For CD4^+^ T cells, this retention has been associated with chemokine receptor-, integrin-, and T cell receptor (TCR)-signaling that affect cell adhesion and motility (8–10). Generally, cell motility depends on the rearrangement of the actin cytoskeleton. In T cells, this is mediated by a crosstalk of several signal transduction cascades including the phosphoinositide 3-kinase- (PI3K) signaling pathway activated via TCR and G-protein-coupled receptors (GPCRs), integrin signaling and the Ras homolog gene family- (Rho-) GTPases (11). It has been shown, that microRNAs interfering with these pathways are able to modulate the motility of lymphocytes (12–14). Thus, microRNAs might contribute to the persistence of proinflammatory Th1 cells in the inflamed tissues, by moderately and coordinately suppressing several genes involved in these signal transduction pathways (15).

Here we have identified the microRNA-31 (miR-31) as regulator of migration in Th1 cells *in vitro*. MiR-31 is selectively and highly expressed in repeatedly activated murine Th1 cells and effector memory Th cells isolated from the synovial fluid of patients suffering from RA. MiR-31 targets a set of genes interfering with PI3K-, Rho-GTPase-, and integrin-signaling. Repeatedly activated Th1 cells expressing high levels of miR-31 showed significantly reduced migration toward CXCL10 compared to Th1 cells activated only once and expressing low amounts of miR-31. Migration of repeatedly activated Th1 cells could be restored by miR-31 inhibition using antagomirs. Expression of miR-31 was dependent on TCR activation, interferon (IFN-) γ and the Th1 master transcription factor T-bet. In contrast, the transcription factor Forkhead box protein O1 (FOXO1) inhibited miR-31 expression directly or by repressing T-bet and IFN-γ.

Thus, miR-31 controls the motility of proinflammatory Th1 cells *in vitro*. The same mechanism might also contribute to the retention of inflamed tissue resident Th cells expressing high levels of miR-31, as observed in RA.

## Materials and Methods

### Mice

C57BL/6, BALB/c, OTII, and *Tbx21*^−/−^ mice were purchased from Charles Rivers and/or bred and kept under specific pathogen-free conditions at the internal animal facility of the DRFZ. Mice were treated conformable to law and euthanized by cervical dislocation. All experiments were approved by the federal state institution “Landesamt für Gesundheit und Soziales” (T0192/10) in Berlin, Germany.

### Cell Culture

CD4^+^CD62L^hi^ (naïve) or CD4^+^ lymphocytes from spleens and lymph nodes of 6- to 10-weeks old mice were isolated and purified as described (16). In brief, CD4^+^ cells were labeled with αCD4-FITC (GK1.5, DRFZ) followed by Magnetic Cell Sorting (MACS) with αFITC microbeads (Miltenyi Biotec). Subsequent purification of CD4^+^CD62L^hi^ cells was achieved using αCD62L microbeads (Miltenyi Biotec). Unless stated otherwise, cells were cultured in “RPMI complete medium” (supplemented with 10% fetal calf serum (FCS), 100 units/ml penicillin, 0.1 mg/ml streptomycin and 10 μM β-mercaptoethanol). To generate Th1, Th2, and Th17 lineage, CD4^+^CD62L^hi^ cells from OTII mice were cultured (1:5) with irradiated (30 Gy), CD90.2 depleted splenocytes (as antigen presenting cells; APCs) in the presence of OVA_323−339_ (0.5 mM) in one of the following polarization media: RPMI complete medium supplemented with IL-12 (5 ng/ml, R&D Systems) and αIL-4 (10 μg/ml, 11B11) for Th1, IL-4 (100 ng/ml, culture supernatant of HEK293 cells transfected with murine IL-4 cDNA), αIFN-γ (10 μg/ml, XMG 1.2), and αIL-12 (10 μg/ml C17.8) for Th2 and TGF-β1 (1 ng/ml, R&D Systems), IL-6, IL-23 (20 ng/ml, R&D Systems), αIL-4, and αIFN-γ for Th17 differentiation (5). For the generation of repeatedly activated Th (rep) cells, viable cells were separated by density gradient centrifugation using Histopaque 1083 (Sigma Aldrich) and reactivated in corresponding polarization medium with freshly isolated APCs every 6 days for three times consecutively (5). For Th1 culture, IL-2 (10 ng/ml, R&D Systems) was added from the second stimulation on. Cells from C57BL/6, BALB/c and Tbx21 were cultured (1 × 10^6^ cells/ml) in Th1 polarization medium with IL-2 and activated with plate-bound αCD3 and αCD28 (3 μg/ml, BD). Unless stated otherwise, cells were removed from the stimulus 48 h post activation and transferred into new cell culture plates. TGF-β1 (5 ng/ml,) or IFN-γ (10 ng/ml, R&D Systems) was added where needed.

### Patient Material

T helper cells were isolated from the synovial fluid of patients suffering from RA or peripheral blood of healthy donors (HC) as described (5, 17). In brief, cells from RA patients were depleted for CD15^+^ cells using MACS. Afterwards, CD3^+^CD4^+^CD14^−^CD45RO^+^ cells were labeled and purified by Fluorescence Activated Cell Sorting (FACS). All gating strategies were performed according to the guidelines for the use of flow cytometry and cell sorting (18). Cells from the blood of HC were separated by density gradient centrifugation using LSM 1077, and purified by FACS as described above. If needed, cells were restimulated with PMA/Ionomycin for 3 h and/or lysed in TRIzol (Invitrogen). All human studies were approved by the Charité ethical committee and the informed consent of all participating subjects was obtained.

### Retroviral Transfection and Transduction of T Cells

Viral supernatant was obtained by calcium phosphate cotransfection of HEK293 cells with plasmids for ectopic expression of a constitutive active form of FOXO1 (pMIT-FOXO1A3) or the empty control (pMIT) (19) together with the retroviral packaging plasmids pCGP and the envelope plasmid pECO. Medium was replaced after 4 h with DMEM complete medium supplemented with HEPES (20 mM). Viral supernatant was collected 24 h−72 h later and stored at −80°C. For the transduction of CD4^+^ T cells from C57BL/6 mice, Th1 polarization medium was removed 36–40 h post activation and the viral supernatant supplemented with polybrene (8 μg/ml) was added followed by centrifugation for 1.5 h at 32°C and 1,800 rpm. After 1 h of incubation at 37°C and 5% CO_2_, viral supernatant was replaced with the former culture supernatant. Forty-eight hours post transduction, cells were labeled with αThy1.1-PE (OX-7, BioLegend) and enriched by MACS using αPE microbeads (Miltenyi Biotec) according to the manufacturer's recommendations. Enrichment efficiency was controlled by flow cytometry (Supplementary Figure 5).

### Inhibition of MiR-31 by Antagomir Treatment

Specific, highly modified, cholesterol-coupled antagomir oligonucleotides (custom synthesized by Dharmacon) (20) were purchased lyophilized and reconstituted as described (21). A miR-31 specific Antagomir-31 (5-mC(^*^)mA(^*^)mGmCmUmAmUmGmCmCmAmGmCmAmUmCmUmUmG(^*^)mC(^*^)mC(^*^)mU(^*^)-3-Chol) and an unspecific Antagomir-SCR control (5-mU(^*^)mC(^*^)mAmCmGmCmAmGmAmUmUmCmAmUmAmA(^*^)mC(^*^)mG(^*^)mU(^*^)-3-Chol) were used. Modifications: 2-O-methyl-ribonucleotides (mN), phosphorothioates in the backbone (^*^) and a cholesterol molecule (Chol) at the 3′end.

Repeatedly activated Th1 (Th1 rep) cells were treated (5 × 10^6^ cells/ml) in serum-free siRNA delivery medium (ACCELL, Dharmacon) containing Antagomir-31 or Antagomir-SCR (1 μM) for 1.5 h at 37°C and 5% CO_2_. Cell suspension was diluted (1:5) with Th1 polarization medium and reactivated with plate-bound αCD3 and αCD28 (3 μg/ml). Inhibition efficiency was assessed by qRT-PCR.

### Inhibition of *FOXO1* and *FOXO3* by siRNA Treatment

A pool of 8 ACCELL siRNAs specific for *Foxo1* and *Foxo3* (4 siRNAs each, Dharmacon) was used to decrease the expression of *Foxo1* and *Foxo3* mRNAs. Th1 rep cells (two rounds of restimulation) (1 × 10^7^ cells/ml) were treated with a mixture of this particular 8 siRNAs (0.25 μM each) or an unspecific siSCR control (2 μM) in serum free siRNA delivery medium (ACCELL, Dharmacon). After 2 h of incubation at 37°C and 5% CO_2_, cell suspension was diluted (1:1) with RPMI medium (final concentrations: 2.5% FCS, 10 μg/ml aIL-4, 5 ng/ml IL-12 and 10 ng/ml IL-2) and cells were activated with plate-bound αCD3 and αCD28 (3 μg/ml).

### Adhesion Assay

A high binding 96-well plate (Corning) was coated with ICAM-1 (R&D Systems) or IgG1 FC (R&D Systems) (10 μg/ml) for 2 h at 37°C. Non-specific binding was blocked with adhesion buffer (HBSS Ca^2+^ Mg^2+^ supplemented with 1% BSA) for 1 h at 37°C. Th1 rep cells were washed twice with PBS, resuspended in pre-warmed, equilibrated adhesion buffer (2 × 10^6^ cells/ml) and starved for 1 h at 37°C and 5% CO_2_. PMA (10 ng/ml), Ionomycin (1 μg/ml) and CXCL10 (100 ng/ml, Immunotools) were added 10 min before the cell suspension was transferred into the coated wells (50 μl/well). Forty-five minutes after incubation and adhesion at 37°C and 5% CO_2_, the plate was washed 4 times with 250 μl warm adhesion buffer using an ELX washer according to the manufacturers recommendations. Adherent cells were detached with ice cold PBS/BSA/EDTA and counted using a MACSQuant (Miltenyi Biotec).

### Transwell Migration Assay

T helper cells were starved in RPMI supplemented with 0.5% fatty acid free BSA (Sigma Aldrich) (migration medium, 4–8 × 10^6^ cells/ml) for 1 h at 37°C and 5% CO_2_. Fifty microliters of the cell suspension, containing 2–4 × 10^5^ cells were transferred onto an ICAM-1 (10 μg/ml) coated membrane (5 μm pore size) in the upper well of a transwell plate (Corning). For transmigration toward the lower well containing 200 μl migration medium supplemented with CXCL10 (100 nM), cells were incubated for 2 h at 37°C and 5% CO_2_. The number of transmigrated cells was assessed by a MACSQuant.

### RNA Isolation and qRT-PCR

Unless stated otherwise, all kits were used according to the manufacturer's recommendations. Total RNA was isolated using ZR RNA MiniPrep^TM^ kit (Zymo Research). Expression values of mature miR-31 (hsa-miR-31, ThermoFisher, assay ID 002279; mmu-miR-31, assay ID 000185) and U6 snRNAs (assay ID 001973) were assessed by qRT-PCR using TaqMan Assays following cDNA synthesis with MircoRNA Reverse Transcription kit. For analysis, expression values of miR-31 were normalized by the change-in-threshold method (2^−Δ*CT*^) to values of obtained from snU6.

Expression values of mRNAs were assessed by SYBR Green based qRT-PCR (Roche) using the following primer pairs: hypoxanthine guanine phosphoribosyltransferase (HPRT) forward 5′-TCCTCCTCAGACCGCTTTT-3′, HPRT reverse 5′-CATAACCTGGTTCATCATCGC-3′, Tbx21 forward 5′-TCCTGCAGTCTCTCCACAAGT-3′, Tbx21 reverse 5′-CAGCTGAGTGATCTCTGCGT-3′, FOXO1 forward 5′-CGGGCTGGAAGAATTCAATTC-3′, FOXO1 reverse, 5′-AGTTCCTTCATTCTGCACTCGAA-3′. Alternatively mRNA was quantified by qRT-PCR based TaqMan Assays (ThermoFisher) using the following assays: ABLIM1 Mm01254316_m1, CD28 Mm01253994_m1, CD69 Mm01183378_m1, CDC42 Mm01194005_g1, EIF4EBP2 Mm00515675_m1, FOXO1 Mm00490671_m1, FOXO3 Mm01185722_m1, HPRT Mm03024075_m1, INFG Mm01168134_m1, KLF2 Mm01244979_g1, LATS2 Mm00497217_m1, LPP Mm00724478_m1, PPP2R2A Mm01317426_g1, PPP3CA Mm01317678_m1, Pri-miR-31 Mm03306874, RAC1 Mm01201653_mH, RHOA Mm00834507_g1, SELL Mm00441291_m1, STK40 Mm00512134_m1, and YWHAE Mm00494242_m1.

In both cases, reverse transcription was performed using the Reverse Transcription kit (Applied Biosystems). For analysis, expression values were normalized by the change-in-threshold method (2^−Δ*CT*^) to values of obtained from *Hprt*.

### Intracellular Staining

Prior to intracellular transcription factor staining, dead cells were labeled with fixable viability dye aqua (Thermofisher). Cells were fixed and stained at room temperature using the FoxP3/Transcription Factor Staining kit (eBioscience) according to the manufacturer's recommendations with fixing and staining times of 1 h each. The following antibodies were used: T-Bet-PE (4B10, BioLegend) and FoxP3-eF450 (FJK-16s, eBioscience).

### Next Generation Sequencing and Determination of mRNA/miR-31 Binding Sites

RNA of Th1 cells after four rounds of restimulation was isolated using the RNeasy Micro kit (Qiagen) and cDNA libraries from a maximum of 100 ng total RNA were prepared using the Tru-Seq Standard Total RNA Library kit (Illumina) according to the manufacturer's recommendations. Quality control was performed with a bioanalyzer using the RNA 600 Pico Kit and RNA with a RIN > 8 was used for library preparation. Paired-end sequencing (2 × 75 nt) was performed on a NextSeq500 Illumina device using the NextSeq500/550 Mid output kit v2 (150 cycles). Raw sequence reads were mapped to mouse GRCm38/mm10 genome using TopHat2 (22) in very-sensitive settings for Bowtie2 (23). The total RNA-sequencing data reported in this paper have been deposited in the Gene Expression Omnibus (GEO) database, https://www.ncbi.nlm.nih.gov/geo (accession no. GSE122218).

A set of 421 conserved putative mir-31 target genes (PTs) was determined using TargetScanMouse 7.1 (24) (413 genes) and augmented by genes found in literature (8 genes) (Supplementary Table 1). To further delimitate the putative targets in Th1 cells the maximal ratio between the coverage of miR-31 binding regions in the 3′-UTR and the median coverage in exons were computed (also see **Figure 2A**). If multiple miR-31 bs within the 3′-UTR of one gene were present, the bs with the highest ratio was included.

### Determination of the miR-31 TSS and Promoter Analysis

The putative transcription start site (TSS) was determined by visual inspection of the RNA-Seq coverage of the genomic region upstream of the mmu-miR-31 stem loop locus (mirBase: chr4:88910557-88910662) and compared to published RNA-Seq data from CD8^+^ T cells (25) using IGV-Browser (26, 27). The respective promoter region was determined visually by the analysis of p300 (28), H3K4me3, and H3K27me3 (29) ChIP-seq data of murine Th1 cells using the Cistrome database (30). Putative binding sites for the transcription factors T-Bet, STAT1, STAT4, and FOXO1 within the promoter region were predicted by ECR Browser (31) based on TRANSFAC professional library V10.2 (32) with a matrix similarity of 0.75. Predicted sites were validated by the respective ChIP-Seq data using Cistrome database (30) for p300 (28), H3K4me3 and H3K27me3 (29), STAT1 (28), STAT4 (33), T-bet (34), and FOXO1 (35).

### Microarray Analysis After miR-31 Antagonism

Microarray experiments were performed according to Niesner et al. (4). In brief, Th1 rep cells were treated with Antagomir-31 or Antagomir-SCR and RNA was extracted 36, 48, and 72 h after antagomir treatment and the RNA quality were controlled as described above. Ten micrograms of the RNA were reverse transcribed and hybridized to Mouse 430_2 (Affymetrix). Raw signals were processed by the affy R package using RMA for normalization to quantify gene expression and MAS5 to determine the present and absent genes (37). Genes present in at least 50% of the Antagomir-31 or Antagomir-SCR treated samples at different time points were defined to be expressed and these genes were used for the follow-up analysis. The Affymetrix microarray data reported in this paper have been deposited in the GEO database, https://www.ncbi.nlm.nih.gov/geo (accession no. GSE122223).

Subramanian et al. (38), Mootha et al. (39) with PT and PT_50_ as gene sets were performed with parameters as shown in Supplementary Table 2d. The significance of higher enrichment of the PT_50_ over the PT set was evaluated by the Welch's test of nominal enrichment scores (NES)s from 1,000 independent GSEA's each using a randomly chosen subset of PT with sizes equal to the size of the PT_50_ set.

For the evaluation of the biological function of miR-31 a GSEA was performed based on KEGG pathways with the same parameters as before. The interaction network was build based on STRING v10.5 using *mus musculus* as host organism. Only validated interactions were included using “low confidence” for experimentally and “high confidence” for database based interactions. The resulting network was arranged and modified manually for interpretation using the Cytoscape application (36). Genes were added to complete TCR- (*Cd3d, Cd3e, Cd3g, Lcp2, Zap70, Nck1*, and *Nck2*) and GPCR- (*Cxcr3, Gnai1, Gnai2*, and *Gnai3*) signaling. Genes without interactions were removed.

### Statistics

Unless stated otherwise, the Mann–Whitney test for unpaired data was used with ^*^p ≤ 0.05, ^**^p ≤ 0.01, and ^***^p ≤ 0.001. Statistical analysis was performed with GraphPad Prism 5.02.

## Results

### MiR-31 Is Upregulated in Repeatedly Activated Th1 Cells and in Synovial Fluid Th Cells From Patients With Rheumatoid Arthritis

**Figure 1:**
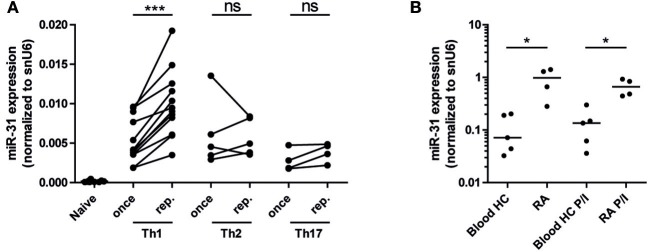
MiR-31 is upregulated in murine Th1 rep cells, and in memory Th cells from the synovial fluid of RA patients. **(A)** MiR-31 expression in once (day 6) and repeatedly (three rounds of restimulation with 6 day intervals) activated Th1, Th2, Th17, and *ex vivo* isolated naive CD4^+^ cells normalized to snU6 determined by qRT-PCR. Each data point represents an independent experiment (*n* = 12 [naive and Th1], 5 [Th2], 4 [Th17]) (Wilcoxon-Test for paired data, ^***^p ≤ 0.001). **(B)** MiR-31 expression normalized to snU6 in CD3^+^CD4^+^CD14^−^CD45RO^+^ T cells isolated from the synovial fluid of patients suffering from RA or blood from healthy control (HC) donors *ex vivo* or after 3 h of restimulation with PMA/ionomycin (P/I) (*n* = 5 RA; *n* = 4 HC) determined by qRT-PCR. Each data point represents an individual donor, horizontal bar: median (Mann-Whitney test for unpaired data, ^*^p ≤ 0.05).

As miR-31 has been shown to be expressed in CD4^+^ (40) and CD8^+^ T cells upon TCR stimulation (25), we aimed to investigate miR-31 expression after repeated antigenic TCR stimulation of murine Th1- cells and in memory Th cells isolated from the inflamed tissue of RA patients. With the rational that Th cells involved in chronic inflammation have a history of repeated restimulation with persistent (auto-) antigens, we once (Th once) or repeatedly activated (Th rep) type 1 (Th1), type 2 (Th2), and type 17 (Th17) lymphocyte subsets (5) and analyzed the expression pattern of miR-31. MiR-31 was expressed in all investigated Th subsets, but was selectively upregulated (3.2-fold) in Th1 rep cells (Figure 1A). CD3^+^CD4^+^CD14^−^CD45RO^+^ memory Th cells isolated from the synovial fluid of patients with RA expressed 8.4-fold (*ex vivo*) and 4.9-fold (after restimulation with PMA/ionomycin) higher levels of miR-31 when compared to their counterparts isolated from the peripheral blood (PB) of healthy subjects (HC) (Figure 1B). Thus, miR-31 was similarly upregulated in inflamed tissue derived Th cells of RA patients and in murine Th1 rep cells, suggesting a comparable function of miR-31 in these cell types.

### A Subset of Putative MiR-31 Target Genes Is Significantly Upregulated After MiR-31 Antagonism

**Figure 2:**
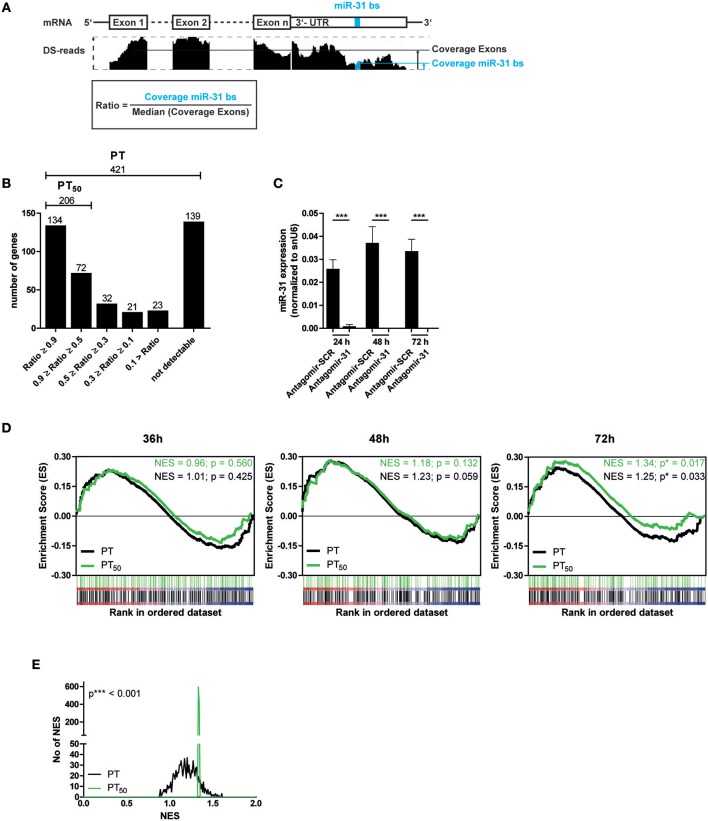
Significant upregulation of miR-31 targets after knock-down of miR-31. **(A)** Schematic overview of the method to determine the fraction of putative miR-31 target mRNA molecules that contain at least one miR-31 bs in their 3′-UTR. Depicted is the coverage (black bars, middle row) of *n* exons and the 3′-UTR (upper row) containing the miR-31 bs (indicated in blue). The ratio is calculated from the median coverage of the exons and the coverage of the miR-31 bs in the 3′-UTR (bottom row). **(B)** 421 putative miR-31 targets were grouped according to the ratio determined in **(A)**. **(C)** MiR-31 expression in Th1 rep cells 24, 48, and 72 h after activation with αCD3/28 and treatment with Antagomir-31 or Antagomir-SCR normalized to snU6 determined by qRT-PCR. Data is shown as mean +SEM, *n* = 11, pooled from five independent experiments (Mann-Whitney test for unpaired data, ^***^p ≤ 0.001). **(D)** GSEA with the PT- and PT_50_- gene-sets and the transcriptome data of Th1 rep cells 36, 48, and 72 h after activation with αCD3/28 and treatment with Antagomir-31 or Antagomir-SCR. Data is shown as enrichment curves with each putative target gene (PT, black; PT_50_, green) in ranked order from most upregulated (left) to most downregulated (right). Nominal *p*-values are depicted in the figure. **(E)** Welch's test of nominal enrichment scores (NES) from 1,000 independent GSEA's each using a randomly chosen subset of PT with sizes equal to the size of the PT_50_ set, *p*-value is depicted in the figure.

Next, we aimed to identify putative miR-31 target genes in Th1 rep cells to investigate the regulatory impact of miR-31. We used TargetScanMouse7.1 (24) in combination with a literature screen to define a list of 421 putative targets (PTs) of miR-31, mainly based on phylogenetic conservation of miR-31 binding sites (bs) and on published targets (Supplementary Table 1). It has been described that activated and proliferating Th cells express mRNAs with shortened 3′ untranslated regions (3′-UTRs) resulting in fewer microRNA target sites (41). To analyze whether the defined PTs contain miR-31 bs within their 3′-UTR in the Th1 rep cells, we performed high throughput sequencing of total RNA (RNA-seq) from resting Th1 rep cells (Figure 2A) and determined the presence of the miR-31 bs in relation to the presence of the protein coding exons. A high ratio of miR-31 bs expression to the median exon expression of the respective transcript would indicate a high probability for miR-31 bs to be present. Of the 421 PTs, 282 were expressed in Th1 rep cells. One hundred thirty-four of them had a miR-31 bs to exon ratio > 0.9, 72 between 0.5 and 0.9, and 76 a ratio < 0.5. For 139 PTs no transcripts were detectable (Figure 2B). Hence, 206 PTs harbor at least one miR-31 bs in more than 50% of the expressed mRNA molecules (PT_50_) in Th1 rep cells (Figure 2B). To validate the predicted target genes of miR-31, we activated Th1 rep cells with anti-CD3- and anti-CD28-antibodies (αCD3/28), concurrently inhibiting miR-31 with a specific antagomir (Antagomir-31) (21). The expression levels of the putative miR-31 targets were quantified by global transcriptome analysis 36, 48, and 72 h following T cell activation and antagomir treatment. MiR-31 expression was significantly reduced by 99% by Antagomir-31 treatment for a period of 72 h after reactivation when compared to Th1 rep cells treated with a control antagomir (Antagomir-SCR) (Figure 2C). No significant enrichment of PT or PT_50_ was observed 36 and 48 h after miR-31 inhibition by gene set enrichment analysis (GSEA). However, after 72 h both PT and PT_50_ gene sets were significantly enriched compared to Antagomir-SCR treated controls (NES 1.25; *p* ≤ 0.017 for PT and NES 1.34; *p* ≤ 0.033 for PT_50_) (Figure 2D), showing even higher enrichments for the PT_50_ subset (*p* ≤ 0.001) (Figure 2E). Of note, 92 of 206 genes showed a positive correlation (rank metric score > 0) after miR-31 knock-down and thus were responsible for the higher enrichment score obtained for PT_50_ (Supplementary Table 2a). By using our novel miRNA target identification approach, which includes the gene expression pattern of the putative targets and the presence of miRNA bs within the their 3′UTRs in a specific cell type, we could increase the identification rate of genes which were affected by miRNA inhibition. Thus, the identified genes are most likely direct target genes of the analyzed miRNA, here miR-31.

### MiR-31 Targets a Set of Motility Related Genes in Th1 Rep Cells

**Figure 3:**
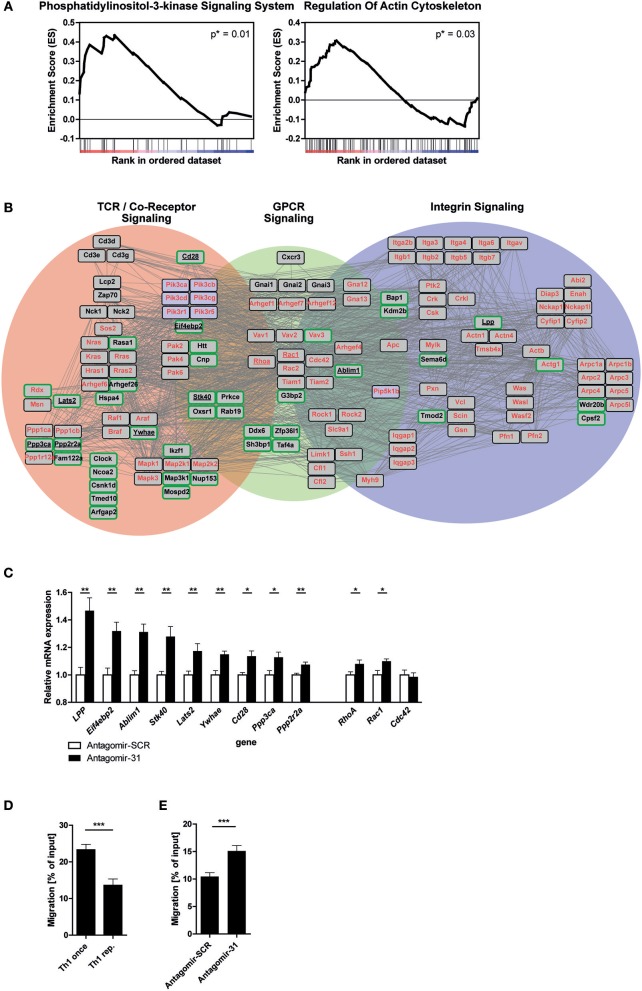
MiR-31 targets a set of genes involved in cytoskeletal rearrangement and miR-31 inhibition increases the motility of Th1 rep cells. **(A)** GSEA of the transcriptome data obtained from Th1 rep cells 72 h after activation with αCD3/28 and treatment with Antagomir-31 or Antagomir-SCR with the KEGG-pathway database (v. 6.0) used as source for gene-sets. Data of two significantly enriched gene-sets is shown as enrichment curves with all genes in ranked order from most upregulated (left) to most downregulated (right). Nominal *p*-values are depicted in the figure. **(B)** Network of validated functional interactions among positively correlated miR-31 targets (green rimmed; Figure 2D) and the genes defining the gene set “regulation of actin cytoskeleton” (red). The resulting network was adapted to T cells (also see methods). Genes without interactions are not included. **(C)** QRT-PCR of target mRNA expression in reactivated Th1 rep cells 72 h after antagomir treatment relative to *Hprt* and normalized to Antagomir-SCR treated control. Data is shown as mean +SEM, *n* = 12 (for *Lats2, RhoA, Stk40, Ywhae*) pooled from four independent experiments, or *n* = 6 (for *Ablim1, Cd28, Cdc42, Eif4ebp2, LPP, Ppp2r2a, Ppp3ca, Rac1*) pooled from two independent experiments (Mann-Whitney test for unpaired data, ^**^p ≤ 0.01, ^*^p ≤ 0.05). **(D,E)** Transwell migration assays with an ICAM-1 coated membrane (10 μg/ml) and CXCL10 (100 ng/ml) in the lower compartment for once and repeatedly activated Th1 cells, 72 h after reactivation with αCD3/28 **(D)** and Th1 rep cells, 72 h after antagomir treatment and reactivation with αCD3/28 **(E)**, assessed by flow cytometry, normalized to inserted cell number. Data is shown as mean +SEM, *n* = 16–18 pooled from four independent experiments (Mann-Whitney test for unpaired data, ^***^p ≤ 0.001).

In order to elucidate the biological function of miR-31 in Th1 rep cells, we performed a GSEA after miR-31 antagonism based on the Kyoto Encyclopedia of Genes and Genomes (KEGG) pathways (42). Here, we focused on 72 h after antagomir treatment, since we observed an exclusive significant enrichment of putative miR-31 targets at this time (Figure 2D). We identified two gene sets as significantly enriched in Th1 rep cells treated with Antagomir-31 as compared to the Antagomir-SCR treated control: “Phosphatidylinositol 3-kinase signaling system” (40 genes, *p* = 0.01; NES = 1.59) and “Regulation of actin cytoskeleton” (108 genes, *p* = 0.03; NES = 1.36) (Figure 3A and Supplementary Tables 2b,c). Since PI3K signaling is part of the network that regulates the actin cytoskeleton (11), we focused on the gene set “Regulation of actin cytoskeleton” in our follow-up analyses. We evaluated the connection of the 92 positively correlating, putative direct miR-31 targets with the 108 genes involved in the regulation of the actin cytoskeleton by creating a network of validated interactions using the Search Tool for the Retrieval of Interacting Genes/Proteins (STRING) (43) (Figure 3B). The resulting network shows the interactions between the PI3K-, Rho-GTPase- and integrin- signal transduction pathways and 41 of the 92 identified putative direct miR-31 targets. In Th1 cells the PI3K- pathway is addressed by TCR- and GPCR-signaling [here as an example CXC motif chemokine receptor (CXCR) 3], which is why we additionally integrated these factors into the network (for detailed description see methods). To validate some putative miR-31 targets from the identified network (Figure 3B) on mRNA level, we measured the expression of 9 candidates after miR-31 antagonism by qRT-PCR. All 9 putative target genes were significantly upregulated 72 h after Antagomir-31 treatment by 10 to 50% compared to Th1 rep cells treated with Antagomir-SCR (Figure 3C). The Rho-GTPases RHOA (Ras homolog gene family, member A), RAC1 (Ras-related C3 botulinum toxin substrate 1) and CDC42 (Cell division control protein 42 homolog) are key mediators of the cytoskeletal rearrangement and thus motility of T cells (11). Compared to Antagomir-SCR treated controls, Th1 rep cells treated with Antagomir-31, showed an upregulation of *RhoA* and *Rac1* by ~8 and ~10%, respectively. In contrast, *Cdc42* remained unchanged (Figure 3C). Thus, miR-31 indirectly regulates the expression of at least two of the central components for cytoskeletal rearrangement via its putative direct targets. Based on the network analysis we hypothesized that miR-31 might affect the adhesion and/or the motility of Th1 cells induced by the TCR-, chemokine receptor- and integrin-signaling.

### MiR-31 Antagonism Increases the Motility of Repeatedly Activated Th1 Cells

To test the potential impact of miR-31 function on the rearrangement of the actin cytoskeleton, we assessed the adhesion of Th1 rep cells restimulated with PMA/ionomycin in the presence of CXC motif chemokine (CXCL) 10 and Intracellular Adhesion Molecule 1 (ICAM-1), i.e., the respective ligands for CXCR3 and Lymphocyte function-associated antigen 1 (LFA-1), both of which are expressed on Th1 rep cells (44, 45). We observed an increase of ~40% in adherent Th1 rep cell number after inhibition of miR-31. Of note, this effect could only be observed in the presence of PMA/ionomycin, ICAM-1 and CXCL10 together (Supplementary Figure 1), which also mimics the milieu of inflamed tissues (46). To examine whether miR-31 also regulates the motility of Th1 rep cells, we tested the migration of Th1 rep through an ICAM-1 coated membrane toward CXCL10 in an *in vitro* transwell migration assay, in which Th1 rep cells migrated ~50% less than Th1 once cells (Figure 3D). This reduced migratory capacity of Th1 rep cells was partly rescued by Antagomir-31 treatment which significantly increased the migration of Th1 rep cells by ~30% compared to Antagomir-SCR treatment (Figure 3E).

The PI3K-, Rho-GTPase- and integrin- signal transduction pathways which we identified to interact with target genes of miR-31 are also linked to T cell activation and thus T cell expansion and effector function. However, no differences in absolute cell numbers and the cytokine production of Th1 rep cells after antagomir treatment and reactivation with αCD3/28 could be detected as compared to Antagomir-SCR treated cells (Supplementary Figure 2).

### MiR-31 Is Induced by TCR Signaling in Th1 Cells

**Figure 4:**
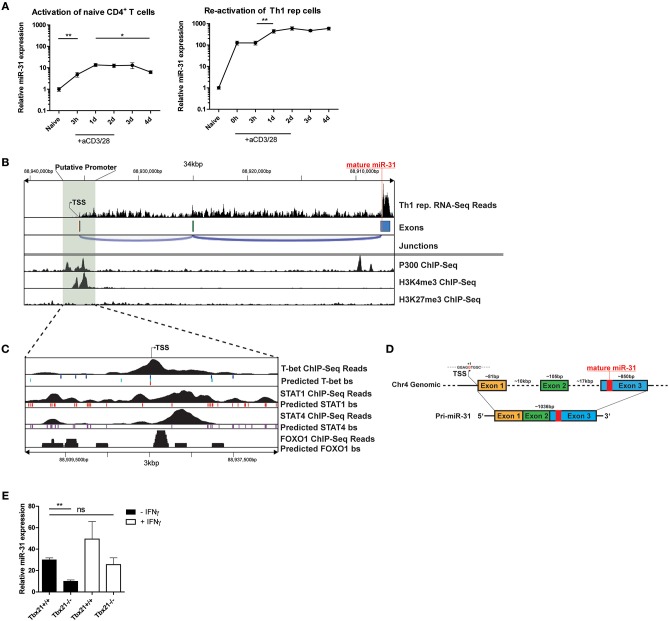
TCR/CD28 induced expression of mir-31 is increased by T-Bet and IFN-γ. **(A)** MiR-31 expression kinetics normalized to snU6 after the first activation of naive CD4^+^ (left panel) or reactivation of Th1 rep cells (right panel) with αCD3/28, presented relative to values obtained from naive CD4^+^ cells *ex vivo*, determined by qRT-PCR. Data is shown as mean ±SEM, *n* = 8–12 pooled from 3 to 4 independent experiments (One-way Anova with Mann-Whitney test for unpaired data, ^*^p ≤ 0.05, ^**^p ≤ 0.01). **(B)** RNA-Seq coverage of the miR-31 gene locus in Th1 rep cells (upper row) and analysis of published ChIP-seq data from Th1 cells for p300 (28), H3K4me3 and H3K27me3 (29) using the Cistrome Browser (lower row). **(C)** Analysis of the putative promoter region as determined in **(B)** using published ChIP-Seq data obtained from Th1 cells for T-Bet (34), STAT1 (28), and STAT4 (33) and from naive CD4^+^ T cells (35), as well as predicted conserved binding sites for these transcription factors obtained from ECR Browser. **(D)** Schematic overview of the murine miR-31 gene locus and the resulting primary transcript as analyzed in **(B)**. **(E)** MiR-31 expression normalized to *Hprt* in naive CD4^+^ cells activated with αCD3/28 in Th1 polarizing conditions for 48 h ± IFN-γ (10 ng/ml), presented relative to values obtained from naive CD4^+^ cells *ex vivo* determined by qRT-PCR. Data is shown as mean ±SEM, *n* = 4–8 pooled from two independent experiments (One-way Anova with Dunn's test for multiple comparison, ^**^p ≤ 0.01).

To investigate the induction of miR-31 during Th1 differentiation, we stimulated naive CD4^+^ T cells with αCD3/28 for 4 days in the presence of Th1 polarizing conditions, i.e., IL-12 and anti-IL-4 blocking antibodies. Compared to naive CD4^+^ T cells, the expression of mature miR-31 was 5-fold upregulated 3 h post activation (p.a.) and further increased 15-fold at 24 h p.a (Figure 4A). Subsequently, miR-31 expression remained stable until day 3 and significantly decreased again by 30% until day 4 p.a. (Figure 4A). In contrast, resting Th1 rep cells already exhibited ~100-fold higher expression than naive Th cells, which did not increase until day 1 after αCD3/28 activation (Figure 4A). Thereafter, Th1 rep cells maintained high levels of miR-31 throughout the remainder of the observation period until day 4 p.a. (Figure 4A). These results suggest that the induction of miR-31 during the initial phase of Th1 differentiation occurs via TCR signaling, whereas in Th1 rep cells additional mechanisms might be involved in the upregulation of miR-31.

### The Primary miR-31 Transcript in Th1 Cells Consists of 3 Exons and Its Genomic Locus Contains Th1 Specific Transcription Factor Binding Sites in the Putative Promoter Region

To understand the transcriptional regulation of miR-31 in Th1 cells, we first determined the transcriptional start site (TSS) for the primary miR-31 transcript using total RNA-seq data from Th1 rep cells. The putative TSS of the murine primary miR-31 transcript is located approximately at chromosomal position mm10:chr4:88,938,478 and spans an intergenic region of ~28.6 kb (Figures 4B,D), which confirms the TSS previously determined by homology analysis of mouse and man (25). The primary transcript expressed by Th1 rep cells consists of 3 exons and has a size of ~1,036 bp (Figure 4D). By reanalyzing Th1 cell ChIP-seq (chromatin immunoprecipitation followed by sequencing) data of p300 (28) and H3K4me3 (29) occupancy using the Cistrome database (30), we observed enrichment of p300 and H3K4me3 binding in close proximity of the putative TSS (Figure 4B). Furthermore, we could identify a CpG island close to this site using USCS Genome Browser (47) (Supplementary Figure 3). To identify transcription factors (TFs), which regulate the transcription of miR-31 in Th1 cells, we performed an *in silico* promoter analysis in a region ±1.5 kb relative to the putative TSS (mm10:chr4:88937246-88940346) using the Evolutionary Conservation of Genomes (ECR) Browser (31). We identified a set of binding sites for TFs important for Th1 differentiation, including Signal Transducer And Activator Of Transcription (STAT) 1, STAT4, T-bet, and FOXO1 (Figure 4C). Reanalysis of additional ChIP-seq data sets showed STAT1 (28), STAT4 (33), and T-bet (34) in close vicinity to the putative TSS of the primary miR-31 transcript (Figure 4C). Since *Foxo1* expression decreased after repeated activation (Supplementary Figure 4), we also analyzed ChIP-Seq data from naive CD4^+^ cells (35) and detected FOXO1-dependent DNA enrichment in the putative miR-31 promoter region. Taken together, we suggest that the expression of mir-31 in Th1 rep cells might be upregulated by a positive feedback loop in a direct or indirect fashion by the activation and induction of STAT1, STAT4, and T-bet as well as the downregulation of FOXO TFs.

### TCR Mediated miR-31 Expression in Th1 Cells Is Increased by T-Bet and the Effector Cytokine IFN-γ

In order to validate that T-bet promotes the expression of miR-31, naive CD4^+^ T cells isolated from Tbx21^−/−^ or wildtype (WT) mice were activated with αCD3/28 for 48 h under Th1 polarizing conditions. The induction of miR-31 expression was diminished by ~50% in activated *Tbx21* deficient Th cells compared to wild type cells (Figure 4E). MiR-31 induction after Th cell activation could be increased to WT levels by adding 10 ng/ml recombinant IFN-γ (Figure 4E). In addition to the *in silico* ChIP-Seq analysis, these data suggest that STAT1, STAT4, and T-bet enforce the expression of miR-31 in differentiating Th1 cells.

### FOXO1 Represses T-Bet and miR-31 Expression in Th1 Cells

**Figure 5:**
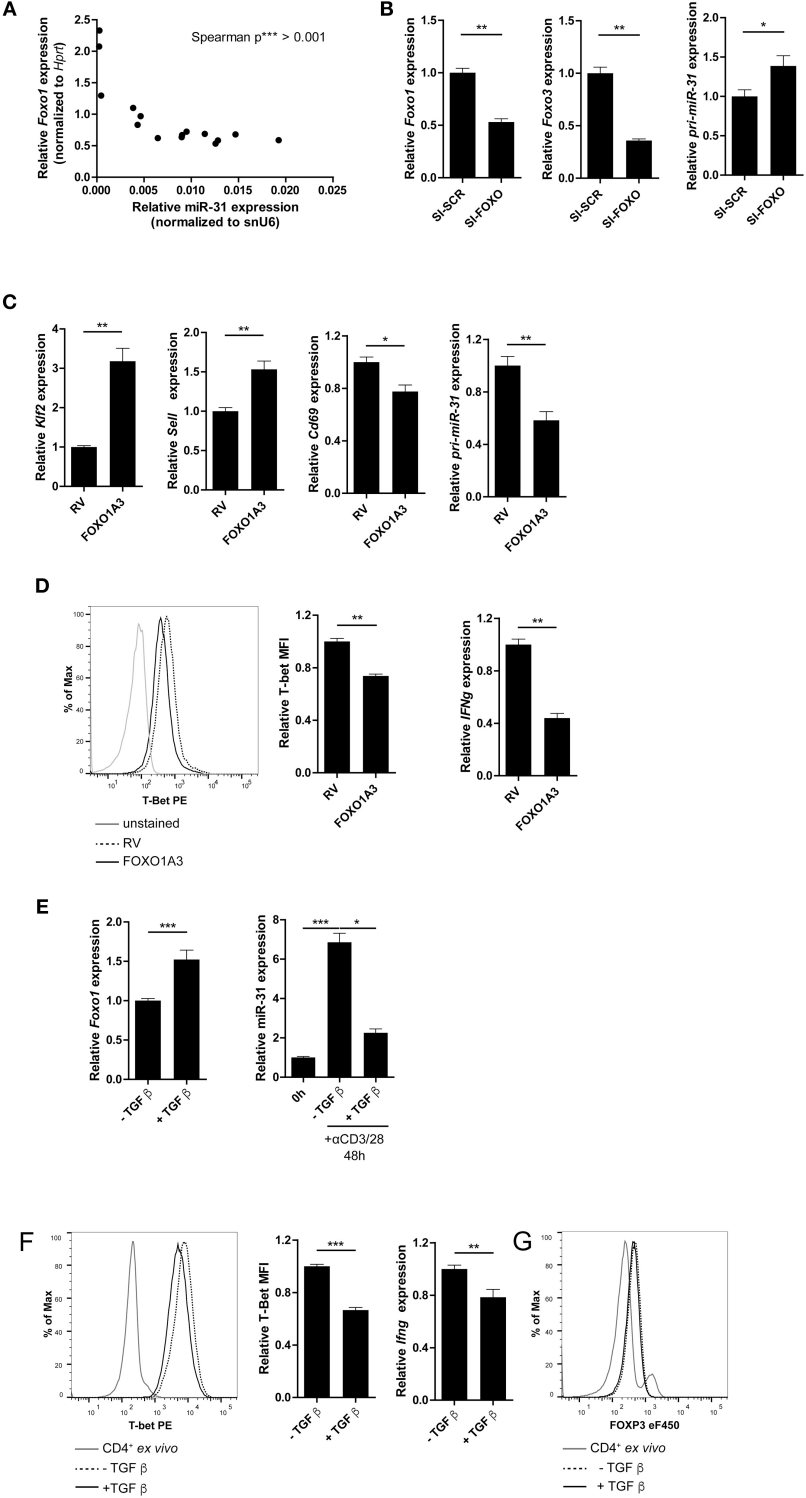
FOXO1 represses T-bet and miR-31 in Th1 cells. **(A)** Correlation between miR-31 expression normalized to snU6 and *Foxo1* expression normalized to *Hprt* determined five times in 6 day intervals from naive to Th1 rep cells (qRT-PCR) (*n* = 15 from one experiment, *p* value is depicted in the figure). **(B)** *Foxo1*, *Foxo3* and *pri-miR-31* expression normalized to *Hprt* in repeatedly (two rounds of stimulation) activated Th1 cells, treated with a pool of 8 siRNAs specific for *Foxo1* and *Foxo3* or a SI-SCR control, analyzed 48 h after siRNA treatment by qRT-PCR, presented relative to the SI-SCR control. Data is shown as mean +SEM, *n* = 6 pooled from two independent experiments (Mann-Whitney test for unpaired data, ^*^p ≤ 0.05, ^**^p ≤ 0.01) **(C)** *Klf2, Sell, Cd69* and *pri-miR-31* expression normalized to *Hprt* in activated CD4^+^ cells transduced 36–40 h post activation with a retroviral vector expressing a constitutive active FOXO1 (FOXO1A3) or an empty control vector (RV). Cells were cultured under Th1 polarizing conditions for additional 48 h. Expression was analyzed by qRT-PCR 48 h post transduction and is presented relative to RV. Data is shown as mean +SEM, *n* = 6 pooled from two independent experiments (Mann-Whitney test for unpaired data, ^**^p ≤ 0.01) **(D)** Representative intracellular protein staining and T-Bet protein expression in the samples analyzed in **(C)**, presented as MFI of T-Bet, normalized to RV assessed by flow cytometry. Data is shown as mean +SEM, *n* = 5–11 pooled from three independent experiments (Mann-Whitney test for unpaired data, ^**^p ≤ 0.01). **(E)** *Foxo1* expression normalized to *Hprt* in Th1 rep cells activated with αCD3/28 under Th1 polarizing conditions for 48 h ±TGFβ, presented relative to values obtained from untreated Th1 rep cells determined by qRT-PCR. Data is shown as mean +SEM, *n* = 9 pooled from 3 independent experiments (Mann-Whitney test for unpaired data, ^**^p ≤ 0.01, ^***^p ≤ 0.001). MiR-31 expression normalized to snU6 in the same cells, presented relative to Th1 rep cells before reactivation assessed by qRT-PCR. Data is shown as mean +SEM, *n* = 9 pooled from three independent experiments (One-way Anova with Dunn's test for multiple comparison, ^*^p ≤ 0.05, ^***^p ≤ 0.001). **(F)** Representative intracellular protein staining of Th1 rep cells activated with αCD3/28 under Th1 polarizing conditions for 48 h ±TGFβ and T-Bet protein expression, presented as MFI of T-Bet, normalized to untreated Th1 rep cells. Data is shown as mean +SEM, *n* = 2–3 pooled from four independent experiments (Mann-Whitney test for unpaired data, ^***^p ≤ 0.001). *Ifng* expression normalized to *Hprt* in the samples analyzed in **(A)**, presented relative to RV. Data is shown as mean +SEM, *n* = 6 pooled from two independent experiments (Mann-Whitney test for unpaired data, ^*^p ≤ 0.05, ^**^p ≤ 0.01). **(G)** Representative intracellular FOXP3 protein staining of Th1 rep cells activated with αCD3/28 in Th1 polarizing conditions for 48 h ±TGF-β.

FOXO1 binds to the TSS of *pri-miR-31* in naïve Th cells and most likely inhibits the expression of miR-31 in these cells. Therefore, we analyzed *Foxo1* expression in Th1 rep cells. *Foxo1* expression in these cells was reduced by ~20% compared to Th1 once cells (Supplementary Figure 4). In addition, *Foxo1* expression negatively correlated with miR-31 expression upon repeated rounds of restimulation in Th1 cells (Figure 5A). To investigate whether FOXO transcription factors including FOXO1, and its functional redundant family member FOXO3, actively repress miR-31, we inhibited *Foxo1* and *Foxo3* mRNAs with a pool of 8 different siRNAs (SI-FOXO) in Th1 rep cells. *Foxo1* and *Foxo3* expression were reduced by ~50 and ~65%, respectively, when compared to control (SI-SCR) treated cells (Figure 5B). This reduction was associated with an increased expression of the primary miR-31 transcript (*pri-miR-31*) by ~40% (Figure 5B, Supplementary Figure 6A shows data for mature miR-31).

The repressive function of FOXO1 on miR-31 expression in Th1 cells was further validated by the ectopic overexpression of a constitutively active form of FOXO1 (FOXO1A3) (19). The FOXO1A3 transduced cells were magnetically enriched by the surface reporter marker Thy1.1 (Supplementary Figure 5) 48 h after transduction. Consistent with published results (48), the expression of the FOXO1 induced transcription factor *Klf2* (Krüppel-like Factor 2) and *Sell*, a gene which encodes for the protein CD62L (L-selectin), were upregulated by 3.2- and 1.5-fold, respectively. In contrast, *Cd69* was down regulated ~20% in cells ectopically overexpressing FOXO1A3 as compared to cells transduced with an empty retroviral vector (RV) (Figure 5C). As expected, the *pri-miR-31* was reduced by ~40% upon overexpression of FOXO1A3 (Figure 5C, Supplementary Figure 6B shows data for mature miR-31), which was associated with a reduced expression of *Ifng* mRNA by ~60% as well as T-bet protein by ~30% as determined by flow cytometry (Figure 5D).

It is known, that the Transforming growth factor (TGF) β stabilizes the expression of total FOXO1 protein (19). Therefore, we induced FOXO1 expression in Th1 rep cells by re-activating the cells in the presence of recombinant TGFβ. Forty-eight hours p.a., *Foxo1* mRNA expression increased by ~50% in the presence of TGFβ as compared to the untreated control, while miR-31 expression decreased by 70% (Figure 5E, Supplementary Figure 6C shows data for *pri-miR-31*). Furthermore, T-bet protein and *Ifng* mRNA were significantly reduced in TGFβ treated Th1 rep cells by ~30 and ~22%, respectively (Figure 5F). This TCR induced miR-31 expression can be blocked by *in vitro* addition of TGFβ, and is most likely due to the induction of FOXP3 (Forkhead-Box-Protein P3) that directly binds within the miR-31 promoter blocking its transcription (40). Notably, the treatment with TGFβ did not cause FOXP3 expression in fully differentiated Th1 rep cells, indicating different control mechanisms for miR-31 expression in different T-cell subsets (Figure 5G). Therefore, the inactivation of FOXO TFs seems to be necessary in order to provide high levels of miR-31 in Th1 rep cells.

## Discussion

**Figure 6:**
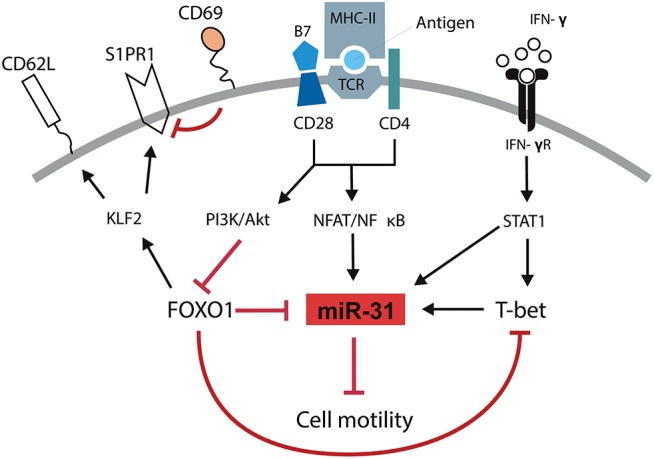
Schematic overview of mechanisms controlling the motility of Th1 rep cells. Antigenic stimulation of the TCR and CD28 leads to the activation of NFAT/NFκB and the PI3K/Akt pathway. IFN-γ induces the activation and expression of STAT1 and T-Bet, respectively. NFAT/NFκB induces the expression of miR-31 which might be amplified by STAT1 and T-bet and reduces the cell motility. PI3K/Akt inactivates FOXO1 which supports the expression of miR-31 either in a direct fashion or by disabling the FOXO1 dependent inhibition of T-bet. Simultaneously, the inhibition of FOXO1 reduces the expression of KLF2, CD62L, and S1PR1 and induces the expression of CD69.

We and others have previously shown that T cells isolated from inflamed tissues of patients with chronic inflammatory diseases are enriched for a Th1 phenotype characterized by the expression of the transcription factor TWIST1 and the secretion of the Th1 signature cytokine IFN-γ (2–4). Interestingly, these cells can be readily found in the inflamed tissues of patients undergoing state-of-the-art immunosuppressive therapies suggesting that they escape from conventional therapeutic interventions (4). This might be due to adaptation of the cells to the inflamed milieu, e.g., by the induction of the anti-apoptotic microRNA miR-148a (5). However, it has remained elusive how these cells are kept in the inflamed tissues, where they mediate and perpetuate chronic inflammation. Here we identified a molecular pathway which controls the motility of Th1 cells with a history of repeated restimulation (Figure 6). The periodic TCR-mediated activation of these cells resulted in an IFN-γ and T-bet dependent upregulation of miR-31, which could be abrogated by FOXO1, a transcription factor expressed in resting Th1 cells. MiR-31 reduced the motility of proinflammatory Th1 cells by regulating the expression of genes which are involved in the rearrangement of actin cytoskeleton downstream of TCR-, chemokine receptor- and integrin-signaling.

To decipher which biological functions are altered following the upregulation of miR-31 in Th1 rep cells we sought to identify its target mRNAs in this particular cell type. Usually, target identification is based on computational prediction of microRNA bs (24). This method delivers a vast number of potential targets including numerous false positive results (49) which have to be excluded experimentally (50). One reason for this could be that most prediction algorithms do not consider 3′-UTR length and, thus, physical presence of predicted miRNA bs. However, it is known that upon stimulation, Th cells in particular shorten their 3′-UTR in order to escape miRNA mediated control (41). By considering the presence of miRNA bs in the 3′UTR, we were able to account for this phenomenon and to increase the identification rate of genes which were affected by the inhibition of miR-31. Interestingly, we observed a significant induction of these genes only after 72 h of activation and Antagomir-31 treatment in restimulated Th1 rep cells. This might be due to the fact that restimulated T cells, in addition to shortening the 3′-UTR, also repress the function of the RNA induced silencing complex (RISC) by ubiquitination and degradation of Argonaute proteins (51) and so escape miRNA mediated control. Taken together, our novel approach for target identification takes kinetic aspects into account which appear to be of great importance for miRNA function in stimulated T cells. To experimentally prove that the identified putative targets are directly regulated by interaction of their 3′UTR with miR-31, repoter assays have to be performed. Indeed, some of the putative targets, e.g., *Stk40* and *Lats*2, were already verified elsewhere using a luciferase reporter assay (25).

Our analysis revealed a modest increase of putative direct or indirect miR-31 target gene expression ranging from 10 to 50% after miR-31 inhibition. These genes are functionally linked to signals downstream of the TCR, chemokine receptors and integrins, as we observed in our network analysis. It is widely accepted that the impact of a miRNA on a specific cellular function increases by targeting several factors that belong to the same biological pathway or protein complex (52, 53). For example, miR-181a targets multiple phosphatases downstream of the TCR and augments the sensitivity of T cells to cognate antigen stimulation (54). Accordingly, by reducing several components of the three pathways mentioned above, miR-31 repressed the motility of Th1 rep cells, most likely by affecting rearrangement of the actin cytoskeleton and cell adhesion. This repression could be attributed to the upregulation of miR-31, as knock-down of miR-31 rescued the motility of Th1 rep cells. Of note, we could not observe a difference in *Cxcr3* expression in these cells (data not shown), which excludes the possibility of disturbed chemokine sensing due to the expression of miR-31. Interestingly, Moffett et al. identified miR-31 as a mediator of CD8^+^ T cell exhaustion by regulating some of the same genes that we also identified as being affected by miR-31 inhibition (25). We assume that these genes also have an impact on TCR-, chemokine receptor- and integrin-signaling in CD8^+^ T cells and thus, miR-31 might also be able to suppress the motility of exhausted cytotoxic T cells. Our results in Th1 rep cells are further corroborated by Fuse et al. showing the KEGG-pathway “rearrangement of actin cytoskeleton” to be enriched for miR-31 target genes and, that a reduced expression of miR-31 is associated with increased migration and invasion of prostate cancer cells (55). Furthermore, miR-31 could also be linked to the migratory behavior of glioma cells (56), ovarian- (57), breast- (58), and gastric cancer cells (59) and hepatocellular carcinoma cells (60). Since TCR mediated activation of the PI3K- and Rho-GTPase- signal transduction pathways also affects the expansion and effector functions of T cells (61), we investigated a possible impact of miR-31 on this. However, knockdown of miR-31 by 99% neither had an effect on the expansion of Th1 rep cells, nor on their cytokine production. This is in line with a study using CD4 specific conditional miR-31 knockout mice and demonstrating that miR-31 has no effect on the proliferation of T cells (40).

Why is miR-31 exclusively upregulated in Th1 rep cells resulting in their reduced motility? Interestingly, we observed a delayed induction of miR-31 in Th1 rep cells as compared to naive CD4^+^ T cells activated under Th1 polarizing conditions. We speculate that the induction of miR-31 in Th1 rep cells is not only dependent on TCR signaling, but also on the master transcription factor T-bet, suggesting that the additional upregulation of miR-31 in Th1 rep cells is dependent on a network of transcription factors active only in Th1, and not in Th2 and Th17 cells. On account of this, we propose that the expression of miR-31 in Th1 rep cells might be sequentially orchestrated via a positive feed-back mechanism involving TCR signaling and the cytokines IFN-γ and IL-12, which activate STAT1, STAT4 (62), and T-bet (5) either independently or synergistically. This is supported by the presence of functional binding sites for the Th1 specific transcription factors T-bet, STAT1, and STAT4 in the proximal promoter region of *pri-miR-31* in Th1 once cells and corroborated by our results obtained from studies with T-bet deficient T cells. With high probability a similar positive feed-back transcriptional mechanism might be active in Th1 rep cells, because these cells produce high amounts of IFN-γ and upregulate T-bet (5). However, it remains to be shown, whether the three TFs, STAT1, STAT4, and T-bet, also bind to the proximal promotor of *pri-miR-31* and to what extent each individual TF contributes to the upregulation of miR-31.

Furthermore, we identified FOXO1 as a suppressor of miR-31 expression in Th1 cells. FOXO1 is highly expressed in resting naive Th cells (63), inhibits cell cycle progression (64, 65), represses T-bet in Th1 cells (66), and regulates T cell homing (67). Ectopic overexpression of a constitutively active form of FOXO1, or *in vitro* TGFβ treatment to induce *Foxo1* expression, led to miR-31 repression and reduced levels of T-bet and the effector cytokine *Ifng*. The repression of miR-31 can be either mediated directly by FOXO1 binding to the promoter of *pri-miR-31*, or indirectly by reducing T-bet and IFN-γ expression. Moreover, we could show that enforced FOXO1A3 expression induced *Sell* (CD62L) and *Klf2* and reduced *Cd69* expression. Taken together, it is possible that the induction of KLF2, CD62L, and CCR7 (67) and a simultaneous repression of CD69 and miR-31 by FOXO1 might license T cells to recirculate to secondary lymphoid organs (48, 68) and to egress from the inflamed tissue (69).

In this study, we used *in vitro* generated Th1 cells, which were subjected to repeated rounds of activation as a model for Th cells derived from inflamed tissues of patients with chronic inflammation (4–6, 70). They mimic inflamed tissue resident Th cells expressing similar levels of the TF TWIST1 (4). In contrast to exhausted dysfunctional CD8+ T cells (71), Th1 rep cells are very efficient in their effector function and induce inflammation in murine transfer colitis (6, 70) and Ovalbumin-induced arthritis (4). In humans, a markedly expanded CD4^+^ T cell population expressing the exhaustion marker PD-1 can be found in the inflamed joints of RA patients. These cells are not anergic or exhausted but can readily provide help to B-cells (72). In line with this observation, we found that the expression of miR-31 was also strongly upregulated in memory Th cells isolated from the synovial fluid of RA patients as compared to their counterparts from the blood, suggesting a different role of this miRNA in CD4^+^ cells in chronic inflammation as compared to exhausted CD8^+^ cells from chronic viral infections (25). In addition, the cells within the inflamed joint receive signals which favor Th1 polarization and miR-31 induction. This is in line with our previous studies showing the upregulation of T-bet and the activity of the IL-12/STAT4 axis in memory T cells isolated from inflamed tissues of patients with autoimmune diseases including RA (4, 5). We hypothesize that the milieu within the inflamed synovium of RA patients favors the induction of miR-31, on the one hand by a repeated auto-antigenic stimulation inducing the expression of T-bet and IFN-γ, and on the other, by IL-7 expression (73) which represses FOXO1 (48), thereby arresting proinflammatory Th cells in the inflamed tissue, where they receive survival signals that counteract immunosuppressive therapies and promote inflammation (4). Therefore, our observation that the miR-31 reduces the motility of Th1 rep cells *in vitro* might reflect the situation in a chronic inflammatory setting. We suggest evaluating the function of miR-31 on Th1 rep cell motility in a chronic inflammatory setting using conditional miR-31 knockout mice (25) or transferring miR-31 expressing or Antagomir-31 treated human Th cells in a humanized model of arthritic inflammation (74) in future studies. In conclusion, reducing miR-31 levels by Antagomir-31 treatment could be a novel approach to mobilize therapy resistant proinflammatory Th1 cells from the inflamed tissues and eventually to resolve chronic inflammation.

## Author Contributions

MB designed the study and performed experiments, analyzed data and wrote the manuscript. CH, PD, KW, AB, MM, CLT, MW, RKA, KL, A-BS, PM, and HR did experiments and/or analyzed data. ML, H-DC, and GAH discussed the results, provided conceptual advice and commented on the manuscript. AR and M-FM designed the study, supervised research and wrote the manuscript.

### Conflict of Interest Statement

The authors declare that the research was conducted in the absence of any commercial or financial relationships that could be construed as a potential conflict of interest.
