# Supplementary material for: MicroRNA-31 Reduces the Motility of Proinflammatory T Helper 1 Lymphocytes
Source: Front Immunol. 2018 Dec 6;9:2813. doi: 10.3389/fimmu.2018.02813 (PMC6291424; doi:10.3389/fimmu.2018.02813)
Supplement: Supplementary file 3 [file Image_1.pdf]

Supplementary Figures

1

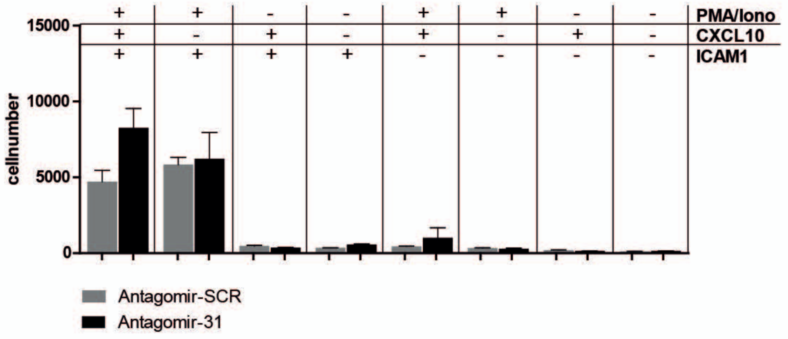

2A

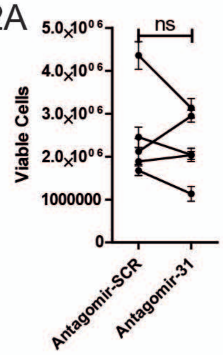

2B

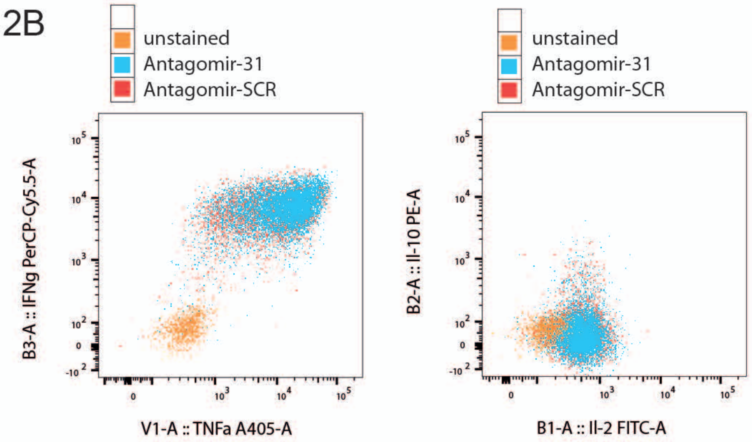

3

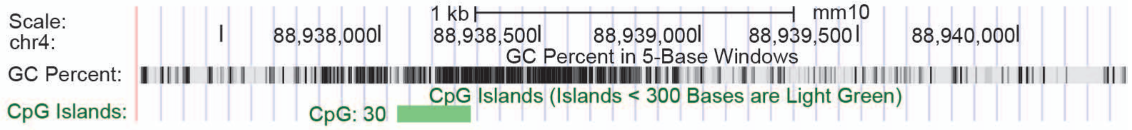

4

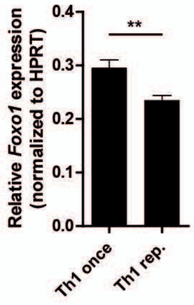

5

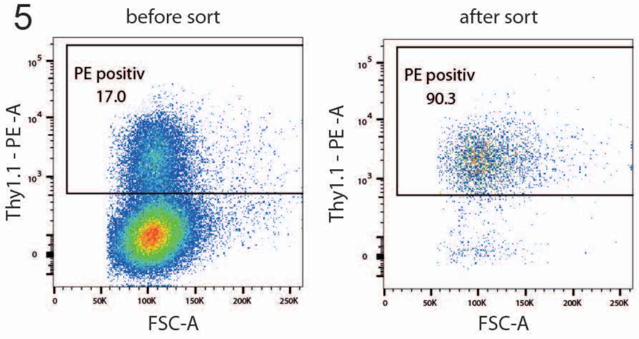

6A

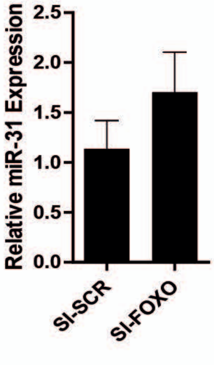

6B

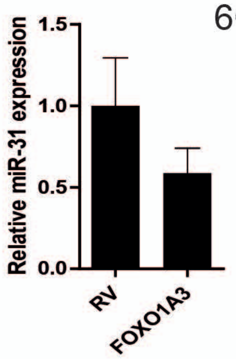

6C

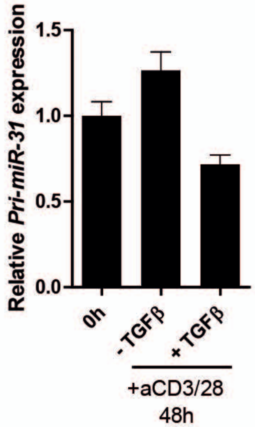

Supplemental Figure legends

Supplementary Figure 1:MiR-31 inhibition increases the adhesion of Th1 rep cells. Th1 rep cells 72h after antagomir treatment and reactivation with αCD3/28 were seeded in an ICAM1 or IgG1 FC (10 µg/ml) coated well for adhesion. Prior to the seeding cells were incubated with PMA/ionomycin and CXCL10. After 45 min, non-adherent cells were removed by an ELX washer and the number of adherent cells was determined using a MACSQuant flow cytometer. Data is shown as mean +SEM, n = 3 from one experiment.

Supplementary Figure 2: MiR-31 inhibition has no significant impact on cell viability and cytokine production in Th1 rep cells. (A) Number of viable Th1 rep cells 72h after reactivation with αCD3/28 and antagomir treatment assessed by flow cytometry. Each data point represents the mean ±SEM of independent experiments with n = 3-6 each (Wilcoxon-Test for paired data, p = 0.6). (**B**) Representative intracellular staining for TNF-α, IFN-γ, IL2 and IL10 of Th1 rep cells restimulated with PMA/ionomycin after antagomir treatment.

Supplementary Figure 3:A CpG island can be found in the putative promoter region. Excerpt from the putative promoter region analysis obtained from UCSC Genome Browser showing a CpG island in close proximity to the putative TSS.

Supplementary Figure 4:Reduced *Foxo1* expression in Th1 rep cells. *Foxo1* expression normalized to *Hprt* in Th1 once and Th1 rep cells (assessed by qRT-PCR). Data is shown as mean +SEM, n = 6 pooled from 2 independent experiments (Mann-Whitney test for unpaired data, $^{**}p \leq 0.01$).

Supplementary Figure 5:Purity of Th1 cells overexpressing FOXO1A3. Representative surface staining of Th1 cells ectopically overexpressing FOXO1A3 with the reporter Thy1.1 determined by flow cytometery.

Supplementary Figure 6: Mature and *pri-miR-31* expression showed similar regulation. (A) Mature miR-31 expression normalized to snU6 in repeatedly (two rounds of stimulation) activated Th1 cells, treated with a pool of 8 SI-RNAs specific for *Foxo1* and *Foxo3* or a SI-SCR control, analyzed 48h after SI-RNA treatment by qRT-PCR, presented relative to the SI-SCR control. Data is shown as mean +SEM, n = 3 pooled from one experiments. (B) Mature miR-31 expression normalized to snU6 in activated $CD4^+$ cells transduced 36-40h post activation with a retroviral vector expressing a constitutive active FOXO1 (FOXO1A3) or an empty control vector (RV). Cells were cultured under Th1 polarizing conditions for additional 48h. Expression was analyzed by qRT-PCR 48h post transduction and is presented relative to RV. Data is shown as mean +SEM, n = 3 pooled from one experiment. (C) *Pri-miR-31* expression normalized to *Hprt* in Th1 rep cells activated with αCD3/28 under Th1 polarizing conditions for 48h ±TGFβ, presented relative to values obtained from untreated Th1 rep cells determined by qRT-PCR. Data is shown as mean +SEM, n = 6-9 pooled from three independent experiments.
